# CMR for evaluation of cardiac function in Type-1 diabetes

**DOI:** 10.1186/1532-429X-18-S1-P150

**Published:** 2016-01-27

**Authors:** El-Sayed H Ibrahim, Jadranka Stojanovska, Scott Swanson, Claire Duvernoy, Rodica Pop-Busui

**Affiliations:** University of Michigan, Ann Arbor, MI USA

## Background

Type 1 diabetes (T1DM) may lead to changes in left ventricular (LV) function and cardiomyopathy in the absence of coronary artery disease or hypertension, possibly due to alterations in sympathetic innervation and myocardial oxidative metabolism and efficiency. The primary aim of this study is to evaluate the association between CMR imaging variables (LV systolic (both global and regional) and diastolic function) and the presence of T1DM at baseline and 3-years follow-up. The secondary aim is to assess the difference of CMR imaging variables in T1DM between males and females.

## Methods

45 T1DM patients (24 females and 21 males; age=34 ± 13 years), and 9 matched healthy controls (HC) (4 females and 5 males; age=34 ± 13 years) were studied with CMR. All T1DM are followed for 3 years, underwent a follow-up CMR exam at 3 years, while adhering to the current standard of care, and CMR at follow-up were available for 27 of the T1DM (15 females and 12 males; age=35 ± 13 years) for T1DM). The CMR exam consisted of cine images (four-chamber and a stack of parallel short-axis slices covering the heart), myocardial grid-tagged images, and transmitral velocity-encoded flow images for measuring left ventricular mass ratio (LVMR=LV mass/LV end-diastolic volume) and ejection fraction (EF), strain and apical-to-basal torsion, and mitral early-to-atrial filling ratio (E/A), respectively. Student's t-test was conducted between patients at baseline and follow-up, between female and male patients, and between patients and HC (p < 0.05 was considered significant).

## Results

**Figure 1:**
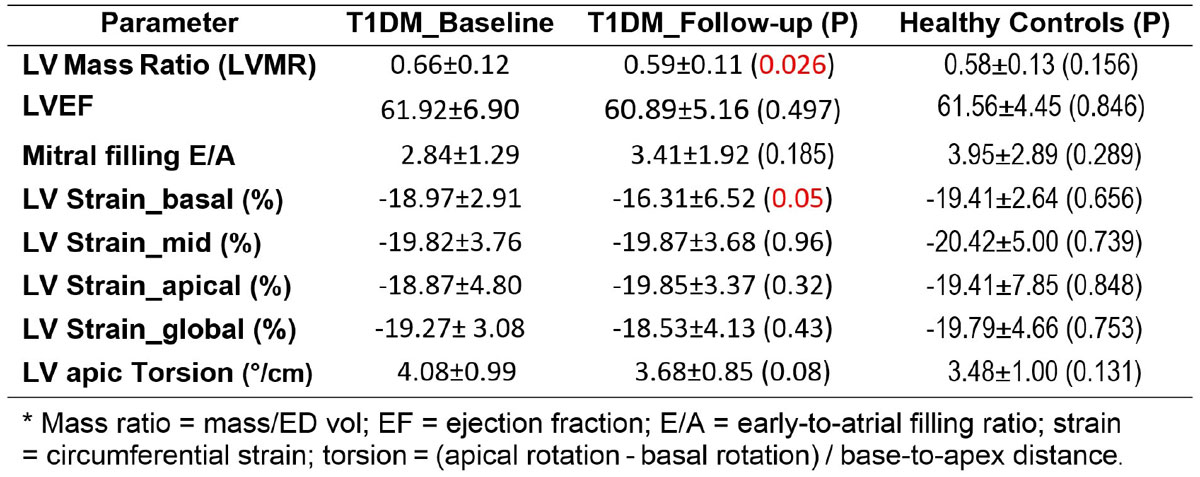
Cardiac functional parameters (mean ± SD) in T1DM (baseline and follow-up) and normals*.

**Figure 2:**
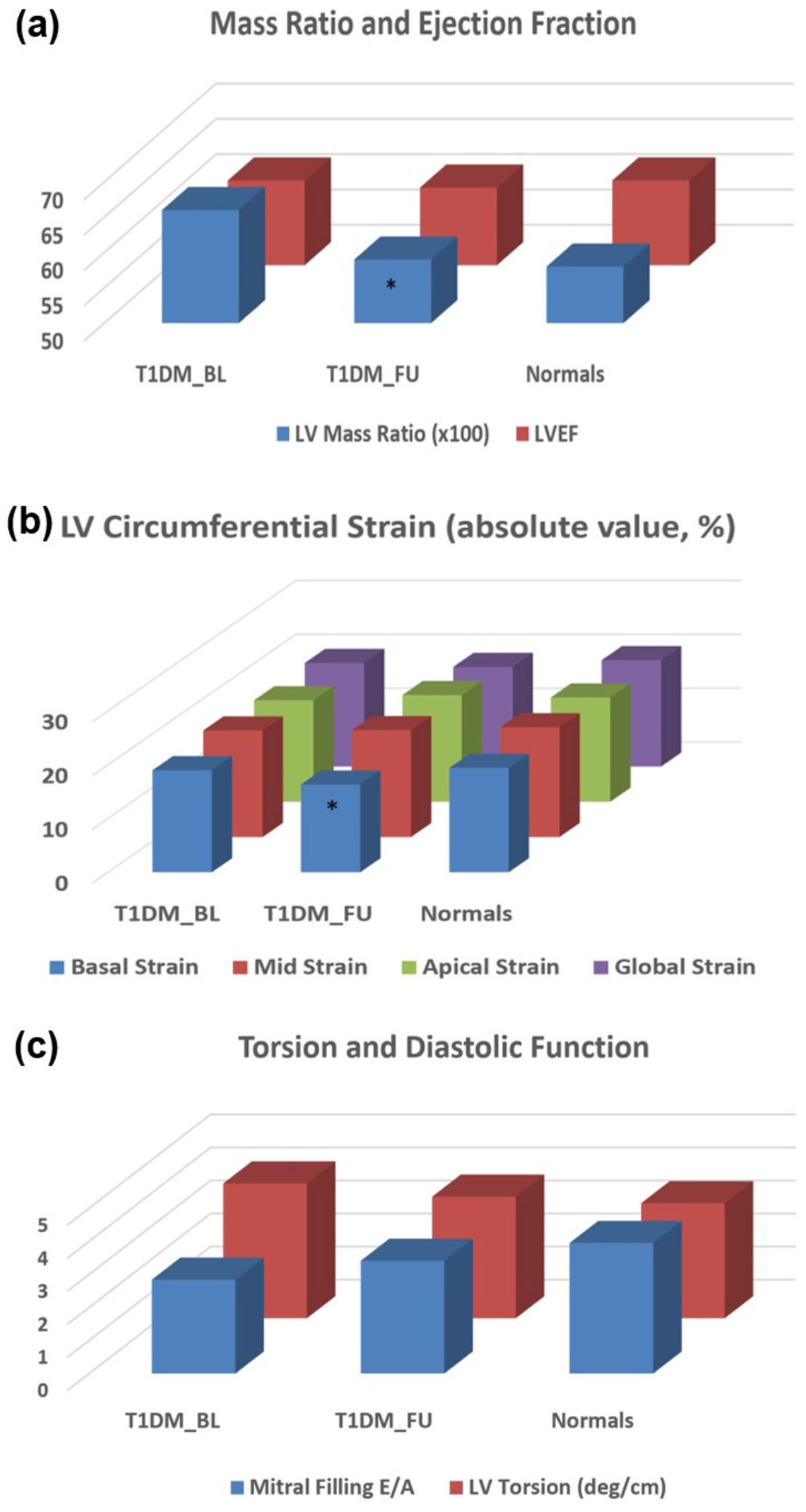
**Cardiac functional parameters in T1DM (baseline (BL) and follow-up (FU) and Normals (HC)**. LV mass ratio = mass/end-diastolic volume; E/A = early-to-atrial filling ratio. * = significant.

There were no differences between T1DM and HC at baseline in any of the measured variables (Figures 1 and 2). In the patient group, only LVMR and basal circumferential strain significantly decreased from baseline to follow-up. Mitral E/A increased, and apical torsion decreased from baseline to follow-up, although these differences were not significant. Among the parameters that showed differences between baseline and follow-up, only LVMR showed significant difference between female vs. male patients, both at baseline (0.69 ± 0.13 vs. 0.62 ± 0.11, p = 0.05) and follow-up (0.53 ± 0.10 vs. 0.68 ± 0.07, p = 0.0001).

## Conclusions

The results demonstrated that at baseline, T1DM showed no significant differences compared to HC. At 3-years follow-up, LV mass (especially in male patients), apical-to-basal torsion, and diastolic function (represented by E/A) normalize toward the measurement ranges in HC, and are accompanied by decreased basal LV strain. In conclusion, CMR is a valuable technique for evaluating global and regional cardiac function in T1DM patients and for following measurements' changes over time. Larger studies with longer follow-up time are needed to better understand the nature of ventricular remodeling in T1DM.

